# Genetic characterization of a novel picornavirus in Algerian bats: co-evolution analysis of bat-related picornaviruses

**DOI:** 10.1038/s41598-019-52209-2

**Published:** 2019-10-31

**Authors:** Safia Zeghbib, Róbert Herczeg, Gábor Kemenesi, Brigitta Zana, Kornélia Kurucz, Péter Urbán, Mónika Madai, Fanni Földes, Henrietta Papp, Balázs Somogyi, Ferenc Jakab

**Affiliations:** 1 Virological Research Group, BSL-4 Laboratory, Szentágothai Research Centre, University of Pécs, Pécs, Hungary; 2 Institute of Biology, Faculty of Sciences, University of Pécs, Pécs, Hungary; 3 Bioinformatics Research Group, Szentágothai Research Centre, University of Pécs, Pécs, Hungary

**Keywords:** Virus-host interactions, Metagenomics

## Abstract

Bats are reservoirs of numerous zoonotic viruses. The *Picornaviridae* family comprises important pathogens which may infect both humans and animals. In this study, a bat-related picornavirus was detected from Algerian *Minioptreus schreibersii* bats for the first time in the country. Molecular analyses revealed the new virus originates to the *Mischivirus* genus. In the operational use of the acquired sequence and all available data regarding bat picornaviruses, we performed a co-evolutionary analysis of mischiviruses and their hosts, to authentically reveal evolutionary patterns within this genus. Based on this analysis, we enlarged the dataset, and examined the co-evolutionary history of all bat-related picornaviruses including their hosts, to effectively compile all possible species jumping events during their evolution. Furthermore, we explored the phylogeny association with geographical location, host-genus and host-species in both data sets.

## Introduction

In the last several decades, bat-related virological studies revealed an increase in the major virus groups highlighting outstanding diversity and prevalence among bats (e.g., *Astroviridae*, *Coronaviridae* and *Picornaviridae*)^1–3^. Although several novel viruses were discovered in these animals worldwide, fewer studies examined the evolutionary patterns regarding these pathogens. Among bat-harbored viruses, members of the *Picornaviridae* family remains neglected with limited available sequence data^4^.

The virus family consists of nearly 80 species grouped into 35 genera, and includes several well-known human and animal pathogens, causing various symptoms ranging from mild febrile illness to severe diseases of heart, liver or even the central nervous system^5^. The family members are small, spherical, non-enveloped viruses, with icosahedral symmetry. The viral genome is a monopartite, linear, polyadenylated positive ssRNA of 7.1–8.9 kb in length, including a single ORF encoding a large polyprotein^6,7^. To date, bat picornaviruses (BtPVs) discovered are associated to the *Mischivirus*, *Hepatovirus*, *Crohivirus*, *Kunsagivirus*, *Kobuvirus* and *Shanbavirus* genus or remain unassigned^8,9^. To the best of our knowledge, *M*. *schreibersii* bats are the primary hosts regarding mischiviruses, which are classified in three distinct species, namely *Mischivirus A*^10^, *Mischivirus B*^11^, and *Mischivirus C*. However, new putative mischivirus sequences were described from both Romanian *Myotis myotis* and *Myotis oxygnathus*. Additionally, other detected BtPVs clustered together with canine and feline picornaviruses^12^ suggests host-jumping events during their evolution.

By understanding virus-host co-evolution history and patterns, disease prediction efforts become more reliable^13^. Coevolutionary studies of major RNA virus groups were performed on coronaviruses and flaviviruses^14,15^. However, BtPVs is a rapidly growing group comprising 8 genera in 2008^16^, and 35 genera in 2017^9^, yet coevolution related studies are still missing.

Bats and viral zoonoses are both neglected and not well researched in Algeria. Therefore, the aim of the current study was drawing the different possible coevolutionary scenarios and assess the degree of association between phenotypic traits and phylogeny, in order to understand more about the virus-host co-evolution within the bat Picornavirus family.

## Materials and Methods

### Sample collection and laboratory procedures

Guano samples were collected in the Jiri Gaisler cave, both the Aoukas and the Melbou caves located in the city of Bejaia, Algeria during 2016 and 2017. Thirty-five fresh bat guano samples were collected from the terrain directly under roosting bats then stored in 2 ml cryo-tubes containing 1 ml of 1x PBS, using sterile dissecting forceps. Samples were next transported in liquid nitrogen and stored at −80 °C until laboratory processes. Nucleic extractions were performed using the Gene JET Viral DNA/RNA Purification Kit (Thermo Scientific), in full accordance the manufacturer’s recommended protocol. Library preparation and sequencing regarding Ion Torrent viral metagenomic analysis were conducted as previously published, including bioinformatics processes, and de novo assembly of sequence readouts^17^. Genome end sequences were amplified with 5′/3′ RACE protocol as described elsewhere^11^, bat DNA barcoding was performed in accordance to Walker and colleagues^18^.

### Sequence data selection

All known bat picornavirus sequences, representing either complete or partial coding sequences were retrieved from GenBank, including the novel mischivirus sequence presented in this study. A total of 70 sequences were analysed (3 kobuvirus, 9 mischivirus, 1 crohivirus, 1 kunsagivirus, 1 sapelovirus, 4 hepatovirus, 1 shanbavirus, 50 unassigned viruses), plus 1 amphibian ampivirus which was represented as an outgroup. The sampling location, collection date, and host genus were listed as indicated in GenBank sequence annotation, and/or the literature^19^ (Supplementary Table S1). The BtPVs sequences were sampled from 26 bat species belonging to 9 genera (Table 1). Sequences provenance hail nearly entirely from Europe n = 35, Asia n = 25, Africa n = 9 and America n = 1. Sequences with unknown hosts were discarded. In order to represent mammalian host evolution, we downloaded both complete and partial mitochondrial cytochrome *b* gene (CYTB)^20^. In reference to the *M*. *schreibersii* bat, we obtained additional CYTB sequences from the Hungarian Natural History Museum in Budapest.

**Table 1:** RdRp genus-specific phylogenetic clusters.

Cluster^a^	Host genus	Host species	Sampling location^b^	RdRp max length
C1	*Eidolon*	1	AM	1198 bp
C2	*Miniopterus*	1	AS	1350 bp
C3	*Myotis*	1	AS	1341 bp
**C4**	***Hipposederos***	**1**	**AF**	**1359** **bp**
C5	Myotis	2	EU	732 bp
**C6**	***Miniopterus***	**1**	**AS, AF, EU**	**1403 bp**
C7	*Rhinolophus*	1	AS	1317 bp
C8	*Eidolon*	1	AF	1356 bp
C9	*Miniopterus*	1	AS	978 bp
C10	*Eidolon*	1	AF	1107 bp
C11	*Miniopterus*	1	AF	1425 bp
C12	*Eidolon*	1	AF	1434 bp
C13	*Coleura*	1	AF	1440 bp
C14	*Rhinolophus*	1	AF	1434 bp
C15	*Myotis*	2	EU	738 bp
C16	*Eidolon*	1	AF	1347 bp
C17	*Vespertilio*	1	AS	1335 bp
C18	*Myotis*	1	AS	1335 bp
C19	*Nyctalus*	1	AS	1332 bp
C20	*Rhinolophus*	3	AS	1344 bp
C21	*Miniopterus*	2	AS, EU	1410 bp
C22	*Nyctalus*	1	EU	744 bp
C23	*Rhinolophus*	2	AS	1383 bp
C24	*Hipposideros*	1	AS	1383 bp
C25	*Rhinolophus*	3	EU, AS	1344 bp
C26	*Ia*	1	AS	1398 bp
C27	*Myotis*	5	EU	744 bp
C28	*Miniopterus*	2	EU, AS	1422 bp

For each cluster host genus, the number of host species, sampling location, and length of the longest RdRp sequence are represented. Abbreviations: ^a^clusters in bold indicate Mischiviruses; ^b^the sampling locations are specified according to large scale area (continents), EU (Europe), AS (Asia), AF (Africa), AM (America).

### Sequence data editing and phylogenetic analysis

Both BtPVs and mischiviruses, and their respective hosts’ sequences were aligned using the MAFFT alignment tool^21^. Sequence length adjustment was acquired in the use of GeneDoc^22^. The size ranged between 1419–1838 bp regarding the P1 region, 1724–2082 bp representative of the P2 region and 343–1404 bp in reference to the RdRp. Host sequences were not modified.

Prior to applying the datasets for phylogenetic reconstruction, we implemented the finest substitution model selection using Mega v6^23^. The GTR + G substitution model was applied for phylogenetic construction based on 3D^pol^ gene from Mischiviruses and their hosts. Additionally, the same substitution model was used to create a tree from P1 region of all BtPVs. Likewise, the GTR + G + I substation model was used to implement phylogeny based on P2 region and 3D^pol^ gene of all BtPVs and their hosts. An amphibian picornavirus *Ampivirus A* sequence was used to root the viral phylogenies^9^, while *Furipterus horrens* CYTB sequence was used as a representative of the outgroup in the host tree. Non-clock Bayesian phylogenetic trees were constructed using MrBayes v3.2.4 software^24^. Each analysis operated for 10 million generations (25% were discarded as burn-in) and sampled every 1000 generations, and the resultant trees were then edited using iTOL^25^.

Pairwise genetic distances were calculated between RdRp nucleotide and amino acid sequences, using the MegAlign pro program (DNASTAR v15.2.0) with uncorrected pairwise distances as a metric, and P distance in MEGA v6^23^.

To assess the temporal signal in the viral data above, a regression method of root-to-tip distances against dates of sampling was implemented regarding the RdRp Bayesian trees, of which, TempEst was used^26^.

### Phylogeny-trait association analysis

Phylogeny-trait statistics were performed, using the association index (AI), parsimony score (PS), and maximum monophyletic clade (MC) index statistics available in the BaTS package^27^. Mischiviruses and all bat picornaviruses (based on the 3D^pol^ gene) were inspected using BaTS software. Both of these exhibited significant bunching by the following character states of interest: bat host genus, species, or sampling location. The values obtained were interpreted according to Parker and colleagues^27^. This analysis compared the posterior distribution of trees regarding our data formerly mentioned, to a null distribution of 1000 trait-randomized trees. The results were interpreted in accordance with Parker and colleagues^27^. Prior, the trace files generated by MrBayes were analyzed in Tracer v1.6^26^, with the aim of discarding the burn-in trees.

### Co-evolution analysis

In order to estimate the virus-host co-divergence scope, we simultaneously analysed picornaviruses (RdRp) and their hosts’ phylogenies along with mischiviruses and their hosts’ phylogenies, all in the operational use of Jane v4.0^28^. It deduces the nature and the frequency of different evolutionary events, by determining the congruence with the least costly reconstructions of the host-parasite connection, using the tree topologies. Thus, the parameters for the entire event costs (co-speciation, duplication, duplication and host switch, loss and failure to diverge) were set to 0, then 1, and after co-speciation, equal to 0 and other events equal to 1 with a population size equal to 100 and 100 generations for both datasets mentioned above.

Tanglegrams for all bat PVs and their hosts, in addition to mishiviruses and their hosts were created using Dendroscope v3-9-5^29^.

### Recombination analysis

To effectively detect recombination, phylogenetic trees generated from various regions of BtPVs genomes (P1, P2 and 3D^pol^) were examined regarding tree structure incongruities. Subsequently, all BtPVs aligned nucleotide sequences were imported in the Recombination Detection Program (RDP 4). Recombination events, parental and recombinant sequences as well as putative breakpoints, all underwent analysis using, GENECONV, BOOTSCAN, GENCONV, SISCAN and MAXCHI methods aligned to default settings^30^, and RDP with internal references only as a parameter.

### Statistical analysis

The strength of the correlation among picornavirus diversity (the number of detected PV clusters) and the number of PV species for each host genus was estimated using the Spearman coefficient (r). The value interpretation was as follows: 0.00–0.39 “weak” correlation, 0.40–0.59 “moderate”, 0.60–0.79 “strong” and 0.80–1.0 “robust”.

## Results

### Detection and genome organization of the novel mischivirus from bats

**Figure 1:**
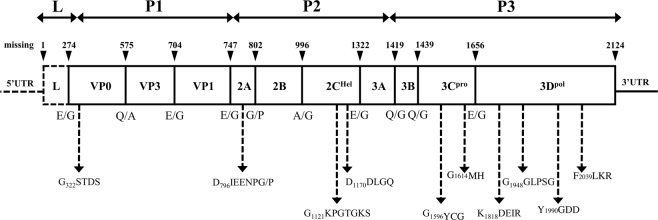
Schematic representation of the novel Algerian BatPV genome organization. 3′ UTR, P1, P2 and P3 regions are included. Also, the putative cleavage sites and the conserved motifs are depicted.

Out of the 35 sequenced metagenomic libraries regarding viral discovery, picornavirus (PV) was found in one library with 1179 reads. Bat DNA barcoding revealed the virus was detected from a *M*. *schreibersii* bat. Based on genome sequence identity level, the novel sequence (MG888045) described in this study is grouped within the *Mischivirus* genus. Nearly the entire genome (6961 nt) was obtained (some 1400 bp are missing, 5′ UTR and the beginning of the L protein). This demonstrates the typical PV characteristic genome organization of UTR [L-P1(VP0, VP3, VP1)-P2(2 A, 2B, 2Chel)-P3(3 A, 3BVPg, 3Cpro, 3Dpol)] UTR-poly(A); and the conservative motifs were very similar to the Hungarian Mischivirus B described by Kemenesi and colleagues^11^. Genome organization pattern, hypothetical cleavage sites and conserved motifs according to the first start codon in the obtained sequence are indicated in Fig. 1.

### Phylogeny and PVs bunching by host and sampling location

According to the Blast results, the novel Algerian sequence shared 85% of nucleotide identity with the Hungarian virus and 73% identity with the Chinese strain. Moreover, it shared between 91–94% identity with shorter sequences available from the Bulgarian tentative mischiviruses.

**Figure 2:**
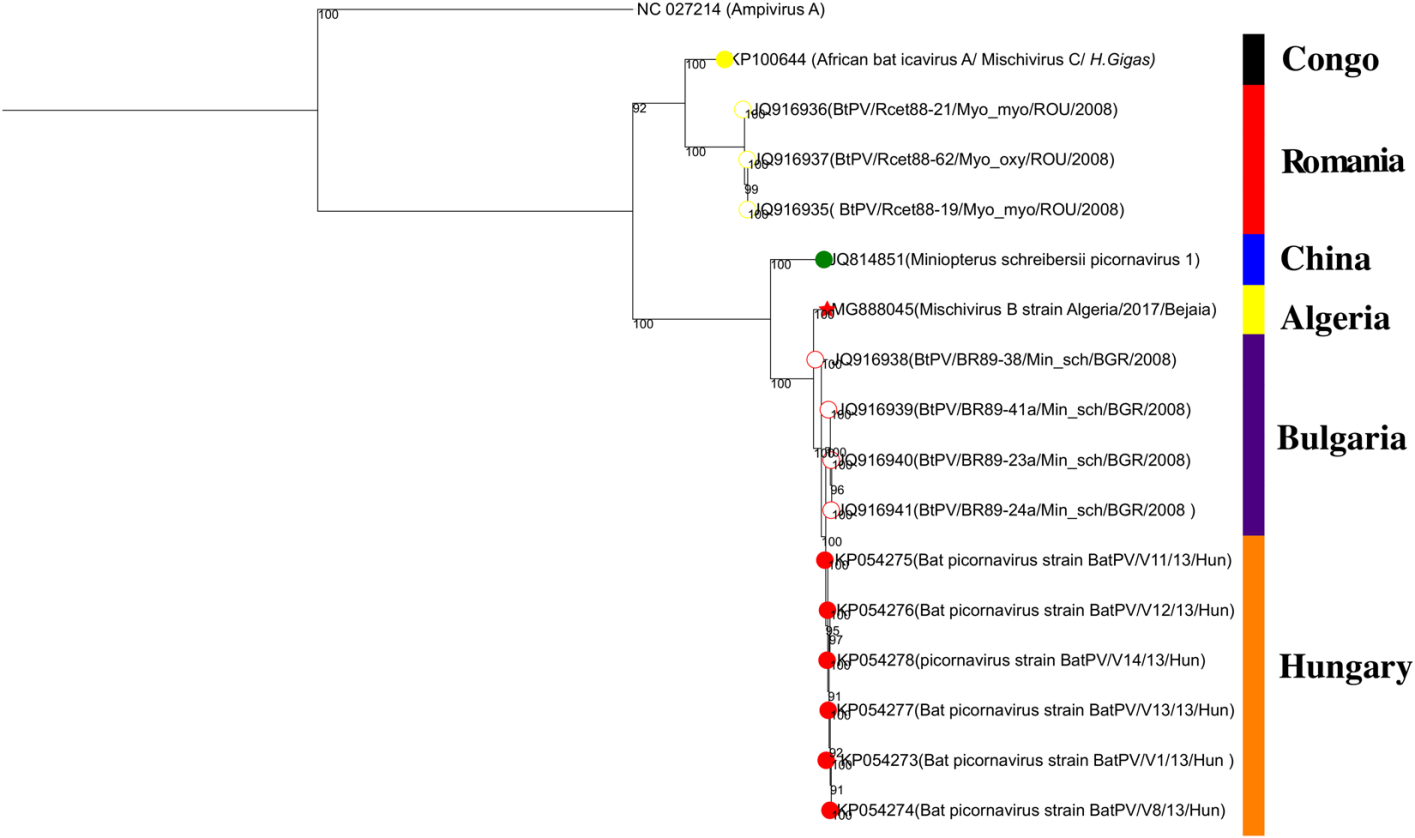
Bayesian interference phylogenetic tree of mischiviruses. The tree exhibits the relationship among the new Algerian mischivirus and other described mischiviruses. The analysis was performed using MrBayes. 3.2.4. ten million generations were performed. Posterior probabilities are indicated at nodes. Branch symbols indicate Mischivirus species. Yellow color: Mischivirus C species, green: Mischivirus A species, red: Mischivirus B species. Solid circles indicate ICTV classified viruses, empty circles indicate the unclassified viruses, and the new Algerian Mischivirus is represented in the use of a star.

The phylogenetic analysis predicated on RNA-dependent RNA polymerase gene (RdRp) of mischivirus sequences (Fig. 2) revealed how the novel Algerian BtPV formed a monophyletic clade together with the Hungarian Mischivirus B sequences^11^; in addition, the Bulgarian and Romanian tentative Mischivirus B^12^, including the Chinese Mischivirus A^10^. The phylogenetic relationship is supported with high posterior probability values (>90%).

**Figure 3:**
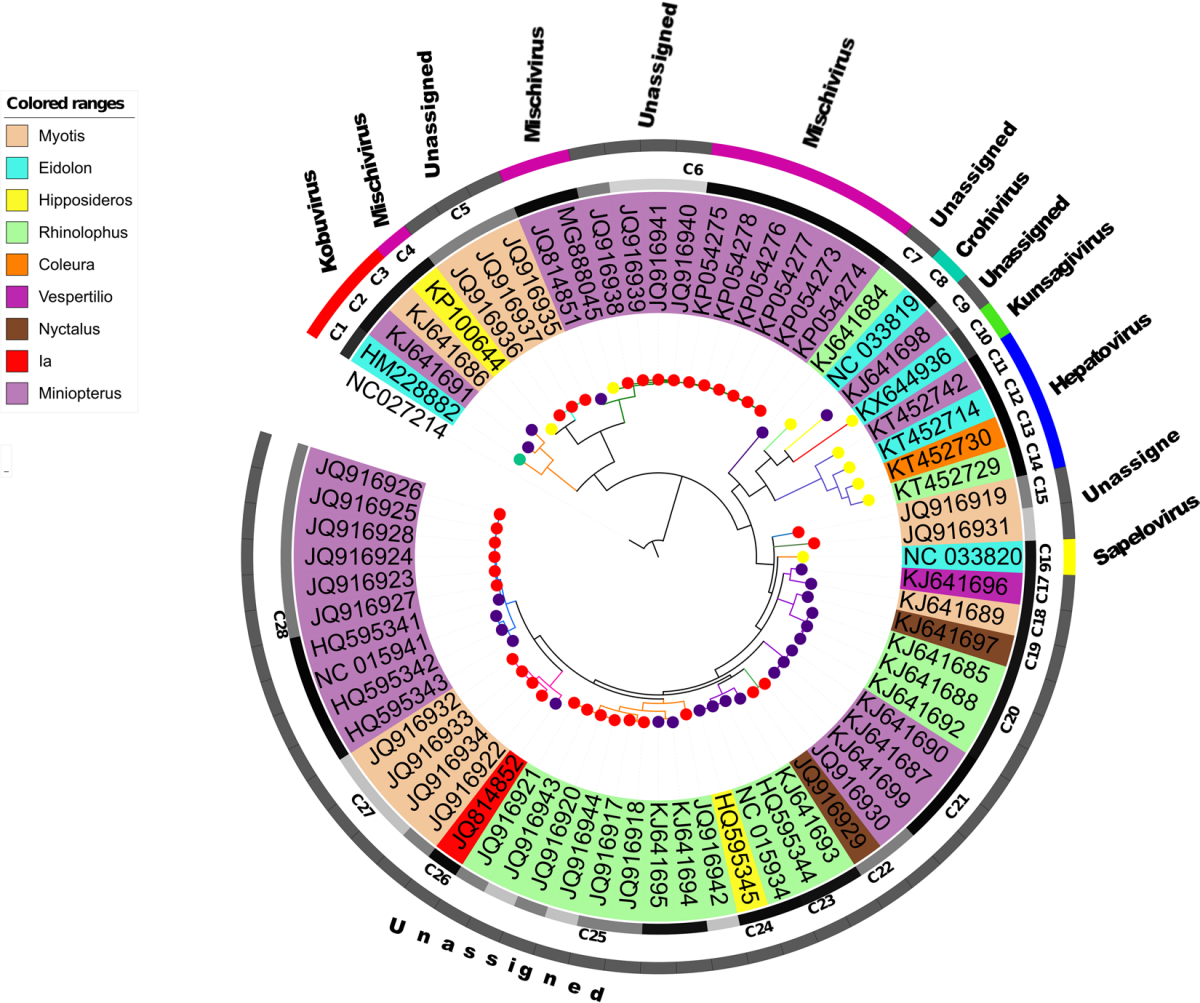
A phylogenetic overview of PVs sequences analyzed. A Bayesian analysis of 70 RdRp sequences, rooted using ampivirus A sequence (NC027214). Branch lengths represent the number of substitutions per site. Genus-specific clusters are colored, based on bat genus. Solid circles represent Large-scale sampling locations, red for Europe, purple for Asia, yellow for Africa, and chartreuse for America. The bar encircling the tree represents the RdRp length range, sequences <500 bp are colored in light grey, sequences between 700 bp and 900 bp in dark grey, and black for sequences >1,000 bp. ICTV virus classification is indicated, if and when available.

An extended phylogenetic analysis to all bat picornaviruses (Fig. 3, Supplementary Fig. S1) exhibited a grouping, related in some areas of the tree with both host genus and large-scale sampling location (continent), while in other areas it is interspersed. Furthermore, bat mischiviruses clustered according to host genus, and sampling location for each virus species (Fig. 2).

Regression analyses (Supplementary Fig. S2) exhibited no association between sampling times and root-to-tip genetic distances, neither for bat mischivirus dataset R^2^ = 0.0829 (Supplementary Fig. 2a) nor for all BtPVs R^2^ = 0.0218 (Supplementary Fig. 2b), and due to this, any molecular clock dating was excluded.

Phylogeny-trait association analysis tests (AI = 0.028, PS = 2) offered statistical support (p < 0.05) regarding the clustering of bat mischivirus (n = 16) when considering host genus. The null hypothesis of no association between phylogeny and host species character trait was accepted based on the association index test (AI = 0.38, p = 0.065), while it was rejected based on the parsimony score test (PS = 3, p = 0). The MC statistic supported the association for both *Miniopterus* (MC = 12, p = 0.001) and *Hipposideros* (MC = 3, p = 0.001) genera, in addition to the *M*. *schreibersii* species (MC = 12, p = 0.001). Regarding the geographical macro area of sampling, the association index (AI = 0.029, p = 0.003) suggested a strong phylogeny-trait association, once the parsimony score (PS = 3, p = 0.23) exhibited no significant relationship. Furthermore, the MC permitted an inspection in each geographic region alone and provided connecting proof for character-trait Europe (MC = 10, p = 0.009). Note how individual traits (single countries, species) consistently provided non-significant results (see Supplementary Table S2).

Furthermore, the same analyses were performed regarding all BtPVs (n = 69), AI and PS provided support (p < 0.05) for associations, when taking into account host genus (AI = 2.25, PS = 21), host species (AI = 3.47, PS = 32), and large-scale sampling area (AI = 0.60, PS = 15). Additionally, the MC statistics representative of each geographical location, host genus, and host species revealed the continents Europe (MC = 10), Africa (MC = 4), and Asia (MC = 5), in addition to the host genera *Miniopterus* (MC = 12), *Myotis* (MC = 4), and *Rhinolophus* (MC = 9), also the species *M*. *schreibersii*, *M*. *myotis*, *R*. *euryale*, *M*. *magnate*, *R*. *sinicus*, *M*. *fuliginosus* were not randomly distributed on the tips of the matching phylogenetic tree (p < 0.05) (Supplementary Table S3). Based on the phylogenetic analyses (Fig. 3) executed using the 69 RdRp gene sequences, 28 different bat genus-specific clusters were identified: three in *Kubovirus*, three in *Mischivirus*, one in *Crohivirus*, one in *Shanbavirus*, one in *Kunsagivirus*, one in *Sapelovirus*, four in *Hepatovirus*, and fourteen clusters in different unassigned viruses. Likewise, we tallied six PV phylogenetic clusters for *Miniopterus* genus, five for *Myotis*, five for *Rhinolophus*, five for *Eidolon*, two for *Hipposideros*, two for *Nyctalus*, one for *Coleura*, and one for *Ia*. Since these BtPVs are related to different host species and sampled from varying locations, the number of RdRp sequences and/or host species within each cluster are different. Strikingly, several of the clusters are composed of a single BtPV RdRp sequence. A very strong correlation was observed among the number of BtPV specific clusters and both its species richness (r = 0.94; p = 0.0001), or geographical sampling area (r = 0.84; p = 0.002). The accurate classification of BtPVs is related to the length of the viral sequences, nonetheless, even short RdRp sequences permitted us to acquire a primary classification of the unassigned sequences.

BtPV sequences are very diverse and pairwise distances calculation resulted in relevant data. The mean nucleotide divergence between sequences from cluster C5 (*Myotis*) and C4 (*Hipposideros*) suggest they are closely related, thus C5 (unassigned) may belong to the genus *Mischivirus* (Table 2 and Fig. 3). The same bat genus can host different virus species which explain the percentage of differences observed among sequences belonging to the same host genus. Mean nucleotide and amino-acid divergence within the same genus were 36% and 54.8% for *Miniopterus*; 45.6% and 60.3% for *Myotis*; 40% and 45.7% for *Rhinolophus*; 55.3% and 78.5% for *Hipposideros*; 44% and 52.3% for *Nyctalus*; 58.7% and 75.5% for *Eidolon*.

**Table 2:** Pairwise genetic distances between and within RdRp clusters.

Cluster^b^	Host genus	Mean within clusters amino-acid pairwise distances	Mean within clusters nucleotide pairwise distances	Mean between clusters nucleotide distances from the closest group^a^	Mean between clusters amino-acid distances from the closest group
C1	*Eidolon*	n/c	n/c	38.8% (0.018) C2	47.7% (0.063) C2
C2	*Miniopterus*	n/c	n/c	38.8% (0.018) C1	47.7% (0.063) C1
C3	*Myotis*	n/c	n/c	27.8% (0.021) C2	40% (0.058) C2
***C4***	***Hipposideros***	**n/c**	***n/c***	***52***.***6%*** (***0***.***021***) ***C3***	***70***.***8%*** (***0***.***053***) ***C3***
C5	*Myotis*	2.1% (0.014)	1.3% (0.003)	25.1% (0.016) C4	29.2% (0.054) C4
***C6***	***Miniopterus***	***13***.***3%*** **(*0***.***023*)**	***8***.***4%*** **(*0***.***006*)**	***39***.***9%***(***0***.***016***) ***C5***	***57***.***8%*** (***0***.***057***) ***C4***
C7	*Rhinolophus*	n/c	n/c	54.5% (0.018) C3	58.5% (0.061) C3
C8	*Eidolon*	n/c	n/c	55.9% (0.019) C5	73.8% (0.053) C5
C9	*Miniopterus*	n/c	n/c	50.1% (0.019) C8	60% (0.062) C8
C10	*Eidolon*	n/c	n/c	56.2% (0.018) C9	72.3% (0.052) C9
C11	*Miniopterus*	n/c	n/c	55.6% (0.018) C7	61.9% (0.054) C8
C12	*Eidolon*	n/c	n/c	36.4% (0.018) C11	43.1% (0.061) C11
C13	*Coleura*	n/c	n/c	35% (0.019) C12	33.8% (0.057) C12
C14	*Rhinolophus*	n/c	n/c	31.1% (0.019) C13	30.8% (0.060) C13
C15	*Myotis*	n/c	n/c	50.5% (0.019) C4	70% (0.051) C11
C16	*Eidolon*	n/c	n/c	43% (0.019) C15	60.8% (0.051) C15
C17	*Vespertilio*	n/c	n/c	37.4% (0.019) C16	50.8% (0.062) C16
C18	*Myotis*	n/c	n/c	30.1% (0.018) C17	41.5% (0.061) C17
C19	*Nyctalus*	n/c	n/c	35.5% (0.018) C17	44.6% (0.052) C17
C20	*Rhinolophus*	10.3% (0.030)	4.1% (0.006)	28.8% (00.17) C19	35.9% (0.057) C19
C21	*Miniopterus*	27.4% (0.032)	19.1% (0.01)	40.7% (0.016) C15	54.2% (0.053) C20
C22	*Nyctalus*	n/c	n/c	28.1% (0.014) C21	43.1% (0.048) C21
C23	*Rhinolophus*	13.3% (0.032)	12.8% (0.01)	37% (0.016) C21	43.6% (0.047) C17
C24	*Hipposideros*	n/c	n/c	6.4% (0.005) C23	43.1% (0.061) C17
C25	*Rhinolophus*	23.5% (0.030)	18.7% (0.01)	35.3% (0.018) C24	44.8% (0.052) C23
C26	*Ia*	n/c	n/c	35.4% (0.017) C25	44.6% (0.063) C19
C27	*Myotis*	n/c	n/c	22.4% (0.018) C26	29.6% (0.053) C26
C28	*Miniopterus*	11.5% (0.026)	9.5% (0.007)	29,2% (0.019) C27	37.7% (0.054) C27

The number of nucleotide and amino-acid differences within and between clusters are shown in percentage with standard error obtained by 1,000 bootstrap. Abbreviations: ^a^distances were calculated among sequences >700 bp; n/c: clusters which have a single sequence; ^b^mischivirus clusters are indicated in italic bold.

### Coevolutionary analyses

**Figure 4:**
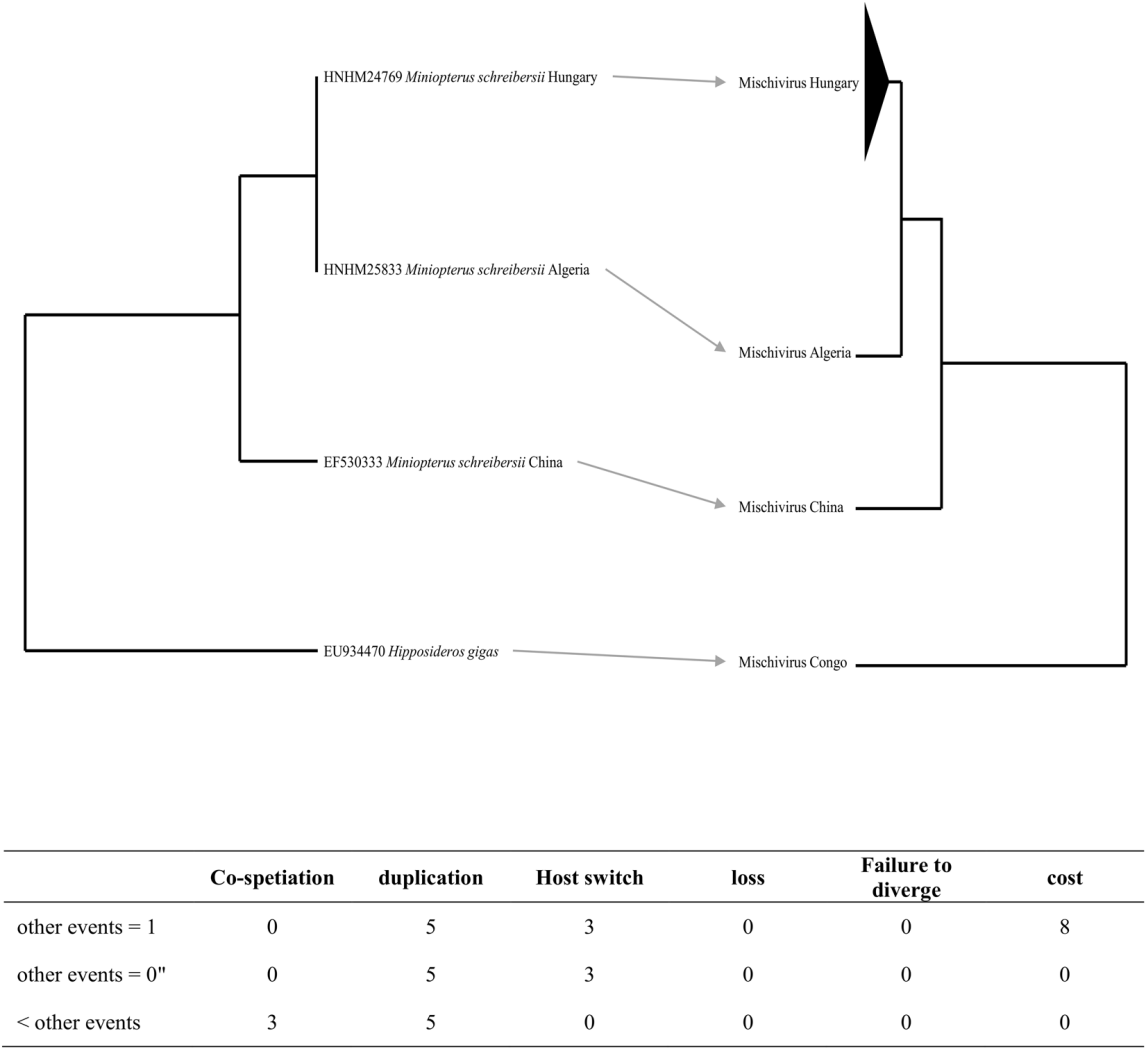
Tanglegram and Jane results of ICTV classified mischiviruses plus the novel Algerian sequence and their hosts. The least costly result is the best evolutionary scenario.

**Figure 5:**
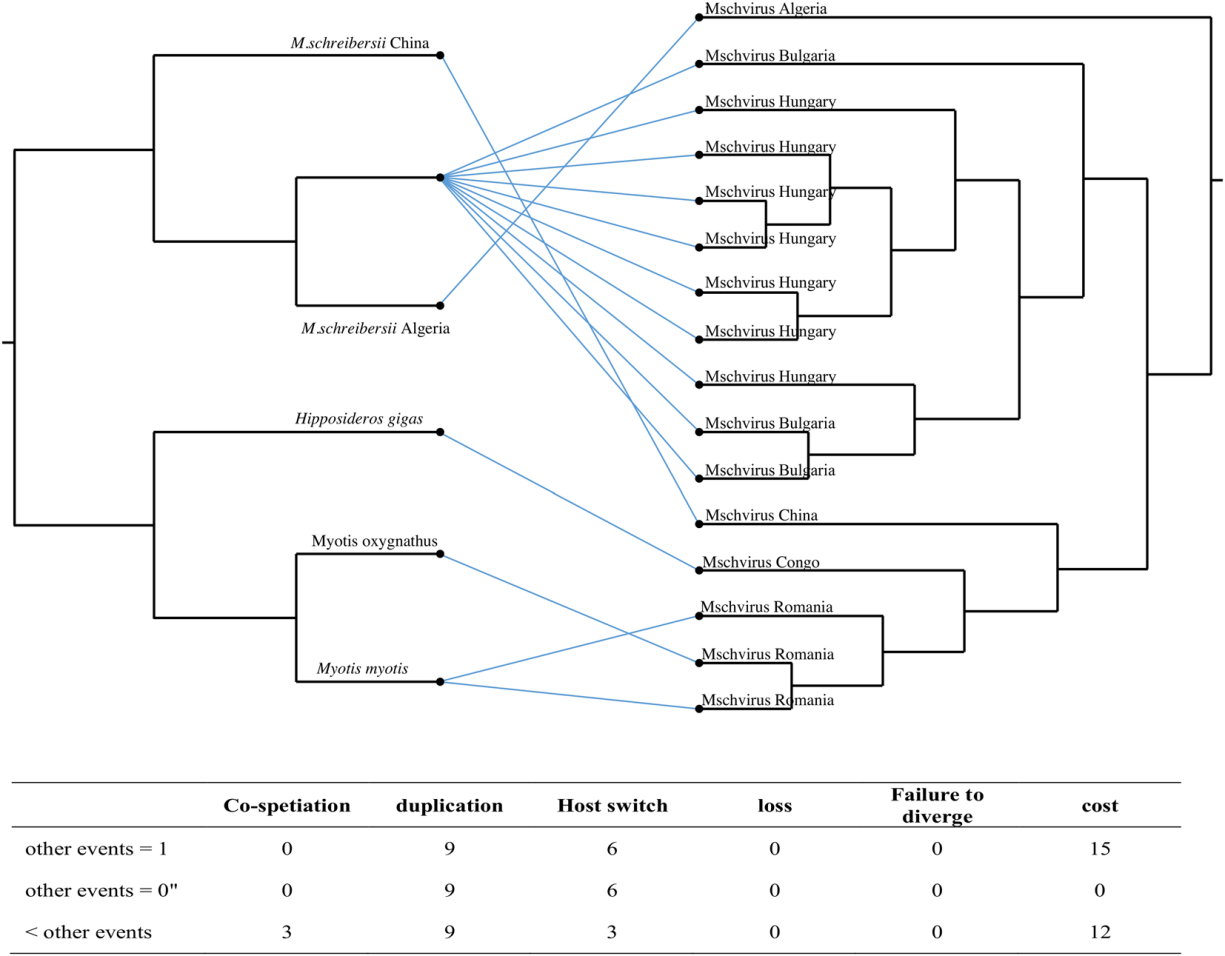
Tanglegram and Jane results of all mischiviruses and their hosts.

In a dataset comprising ICTV classified mischiviruses and our sequence, topological similarities were observed mischiviruses and their hosts’ phylogenies (Fig. 4) suggesting first, a co-divergence evolutionary scenario, which interestingly, was not supported by reconciliation analysis (using Jane), when considering the least costly event. When taking into account both all putative and classified mischiviruses, incongruence topology was observed, in addition to reconciliation analysis which revealed how host-jumping events were involved in the evolution of mischiviruses (Fig. 5).

When extending the analysis to all BtPVs and their hosts’ phylogenies, the history of their evolution was explained by more host-jumping events than co-speciation events, generating incongruent tree topologies (Supplementary Fig. S3). Jane analysis displayed more duplication and host switch events than co-divergence events, independently of co-divergence costs (Table 3). The least costly events represented the best evolutionary scenario, Jane results helped us to determine hypothetic donors and receptors in cross-species transmission events.

**Table 3:** Reconciliation analysis for all bat PVs. Co-phylogenetic reconciliation analysis (Jane) of all bat PVs sequences and their hosts displaying the frequency of different evolutionary scenario. Abbreviation: ^a^least costly events (cost = 0).

	Co-speciation	Duplication	Host switch	Loss	Failure to diverge	Cost
Co-speciation = other events = 1	2	27	39	4	1	73
Co-speciation = other events = 0^a^	0	27	41	5	1	0
Co-speciation < other events	5	25	38	4	1	68

### Recombination analysis

**Figure 6:**
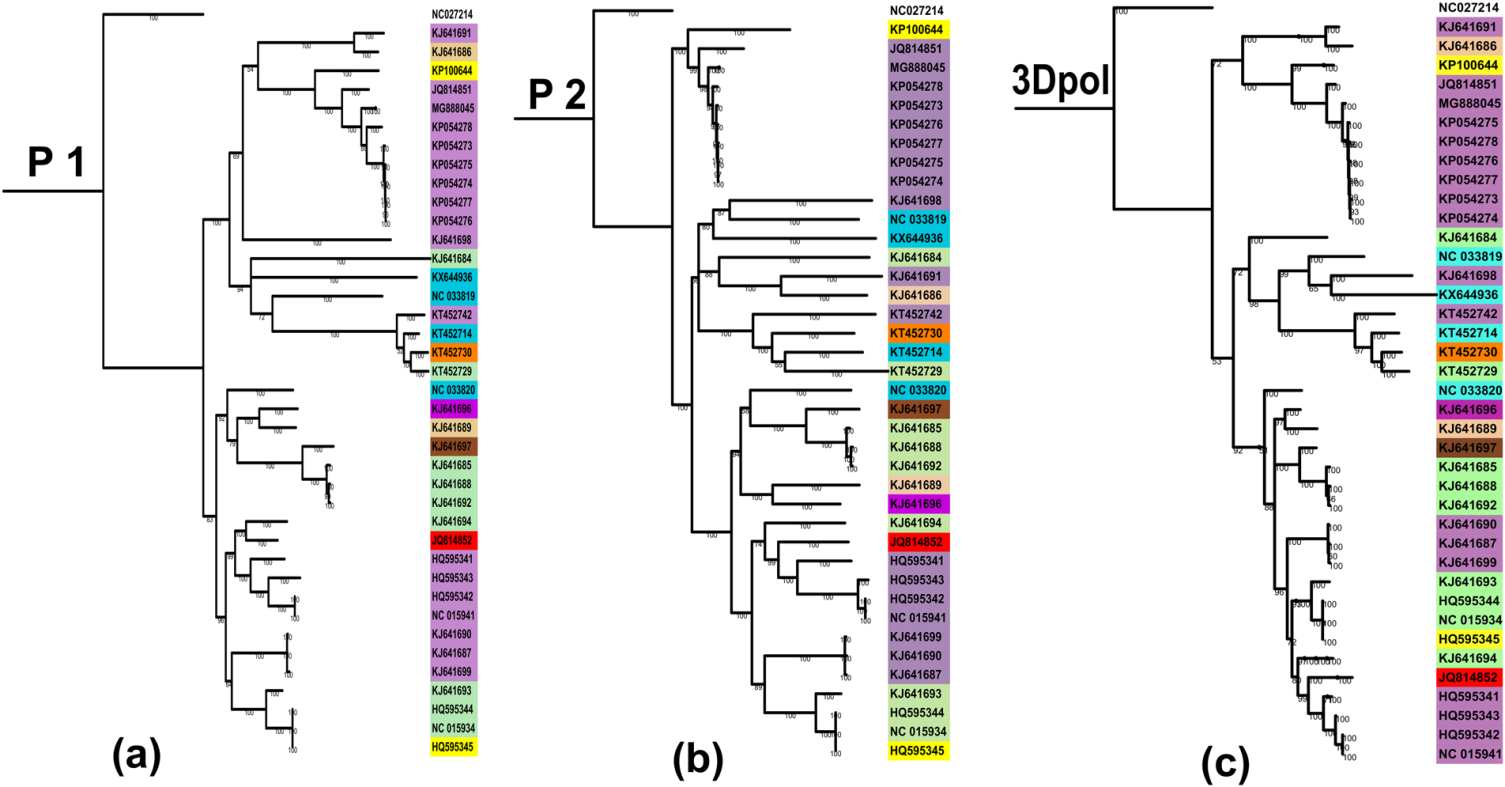
(**a**) Bayesian phylogenetic reconstruction of P1 region for all BtPVs genomes included in this study. (**b**) Bayesian phylogenetic reconstruction of P2 region for all BtPVs genomes included in this study. (**c**) Bayesian phylogenetic reconstruction of RdRp region for all BtPVs genomes included in this study. Ampivirus A sequence (NC027214) used as an outgroup for the three viral phylogenetic trees. Genus-specific clusters are colored, based on bat genus. Recombination may be reflected in tree structure incongruities between phylogenetic trees (**a**–**c**).

The phylogenetic trees built from P1, P2 and 3Dpol regions of BtPVs genomes displayed discordances in their structures indicating potential recombination events (Fig. 6a–c). Out of the 69 recombination events detected with the RDP 4 program, only 11 unique putative recombination events identified with two or more methods were retained (Table 4).

**Table 4:** Summary of recombination events detected by six algorithms within the Recombination Detection Program RDP4. ND: not detected.

Recombinant sequence	Breakpoint position in recombinant sequence		Parental sequence(s)		Score for the detection methods in RDP (P value)					
	Begin	End	Major	Manor	RDP	GENECONV	Bootscan	Maxchi	Chimaera	SISscan
HQ595342	581	624	HQ595343	KJ641694	8,2 × 10^−7^	ND	ND	ND	ND	3,1 × 10^−5^
KP100644	4228	4329	MG888045	Unknown	ND	ND	ND	5,8 × 10^−4^	3 × 10^−21^	ND
KJ641696	2847	3015	KJ641697	NC_027214	2,6 × 10^−2^	ND	ND	ND	ND	ND
KP054278	3703	6850	Unknown	KP054276	ND	ND	1,6 × 10^−19^	5,1 × 10^−15^	4,7 × 10^−3^	9,2 × 10^−5^
HQ595341	332	715	NC_015941	KJ641693	ND	ND	ND	7 × 10^−8^	1,7 × 10^−6^	9,1 × 10^−7^
KT452729	704	885	KT452730	KT452714	6,8 × 19^−9^	8 × 10^−4^	4,7 × 10^−5^	5,8 × 10^−7^	3,1 × 10^−8^	1,7 × 10^−7^
KT452742	4232	4579	Unknown	KT452730	2,1 × 10^−6^	4,5 × 10^−3^	ND	4 × 10^−4^	4 × 10^−4^	4 × 10^−4^
KT452714	5407	5509	KT452742	Unknown	ND	ND	3 × 10^−2^	4,2 × 10^−4^	ND	1 × 10^−2^
HQ595341	4972	5185	KJ641694	NC_033820	ND	ND	ND	5,5 × 10^−3^	ND	1 × 10^−11^
KP100644	3090	3185	KP054278	Unknown	ND	ND	ND	4,3 × 10^−2^	ND	2,1 × 10^−2^
HQ595343	0	6615	JQ916923	NC_015941	2 × 10^−8^	5 × 10^−7^	ND	7 × 10^−9^	6,6 × 10^−9^	5,8 × 10^−13^

## Discussion

Numerous factors (such as geographical, demographical or ecological) likely influence the occurrence of spill-over events and act as driver factors in viral evolution^31,32^. Destruction of the natural habitat of bats worldwide facilitated the urbanization of several dedicated species or simply established more opportunities for human-bat contact events^33^. This constitutes novel possible factors regarding viral emergence as already seen with coronaviruses^34^ and rabies^35^. Understanding the evolutionary mechanisms of BtPVs is a prominent direction of research.

In addition, the zoonotic potential of all documented BtPVs is clearly unidentified yet and fairly misunderstood^36^. Although animal originated PVs were previously exemplified as potential zoonotic agents, as in the case of the encephalomyocarditis virus, which was clearly revealed through experimental infections on human tissues and primary cell cultures^37^.

Understanding viruses and their hosts’ coevolution is crucial to show up the evolutionary character and understand potential disease emergence factors^38,39^. Thus far, several phylogenetic and systematic evolutionary studies were performed with regards to the *Picornaviridae* family, as the virus members of this family are increasingly discovered^40,41^. However, little is still relatively unknown in reference to the virus-host coevolution patterns for this group. In this study, we describe the first picornavirus from Algerian bats, and we include this novel sequence in detailed coevolutionary analysis, focusing on BtPVs.

Picornaviruses exhibit a high genetic diversity (quasi-species) similar to other RNA viruses^42,43^, and a broad geographical spectrum in *Chiroptera* order. Moreover, a positive relationship was previously described between the viral richness and geographical distribution of bats^44,45^. Similarly, in the present study, a positive correlation between PV diversity, species richness, and geographical distribution was revealed. Furthermore, more than one virus cluster and larger viral clusters were obtained for genera in which more species have been sampled in a large geographical range.

BtPVs originating from the same host genus were very diverse and not closely related, hence, both BtPVs and bat mischiviruses did not demonstrate any specificity, whether on the species level nor on the genus level. Likewise, for tortoise picornavirus^40^ and contrariwise for coronaviruses^46^.

Several BtPVs clusters possess a single viral sequence whereas several sequences were considerably short. For instance, mischiviruses displayed three host genus related clusters. *Miniopterus* genus virus related cluster comprising the Asian mischivirus sequence (8468 bp), the new Algerian sequence (6,961 pb), the six Hungarian sequences (6,855 bp), and four partial 3Dpol Bulgarian sequences (three sequences 343 bp, one 983 bp). *Myotis* genus cluster composed of three Romanian partial 3D pol sequences (993 bp), while the *Hipposideros* genus cluster consisting of a single Mischivirus C sequence from the Congo (8096 bp). Both the number and the length of sequences often limit the analyses. By way of illustration through the use of BaTS software if and when the number of sequences regarding a character trait is lower than three, the result is declared insignificant (case of America). Therefore, short sequences may likely be misclassified.

According to Lewis-Rogers and Crandall^43^, PVs and their hosts evolve through host-jumping events and not via co-speciation. Chiropteran order was not included in the previous study. The main finding of the present study was the frequency regarding host-jumping events occurring in the evolutionary history of BtPVs, and reflected as incongruence between the virus and the host phylogeny. Furthermore, we could observe nearly all bat genera host phylogenetically divergent viruses and an absence of species specificity. According to studies performed on coronaviruses^14^ it may likely be due to multiple introductions of PVs, this supposition is coherent with the detection of highly related PVs in humans and different animal species, such as the aichivirus 1 in humans^47^ canine kobuvirus 1^48,49^, murine kobuvirus 1^50^, feline kobuvirus^51,52^, all belonging to the virus species Aichivirus A in the genus *Kobuvirus*. Moreover, the *Mischivirus* genus was supposedly restricted to the *M*. *schreibersii* bat, however, later it was associated to the *H*. *gigas*, *M*. *oxygnathus*, *M*. *myotis* and surprisingly, to the foxhound^53^.

Cross-species transmission event occurrence is multifactorial, nevertheless, we concluded in some cases of BtPVs, sympatry may increase host-jumping events. Co-roosting of Myotis and Miniopterus bats may likely explain the detection of closely related kobuvirus associated among these bats^14^. The migratory ability of Miniopterus bats with the longest distance of 883 km recorded in Europe, which possibly eases the spread of viruses among bat populations spread out in geographically distant areas. For example, *M*. *schreibersii*, originating from Europe and Algeria, which possess different geographical distribution and share the same virus species *Mischivirus B*^54^.

Despite the fact in which no interaction is known regarding *M*. *oxygnathus* and *H*. *gigas* in accordance with their ecologies, hypothetical cross-species transmission events were detected among these two species. The length of the viral sequence obtained from *M*. *oxygnathus* (993 bp) is a limitation regarding accurate classification. Based on the phylogenetic analyses (Fig. 2), the putative mischivirus sequence obtained from *M*. *oxygnathus* and *M*. *myotis* were closely related to Mischivirus C, identified from *H*. *gigas*. Although, they revealed an identical branching pattern upon the structure of the phylogenetic tree as Mischivirus A from *M*. *schreibersii* sampled in China and Mischvirus B from Algeria and both Bulgaria and Hungary, indicating BtPVs from Romania may not belong to Mischivirus C. Furthermore, it has been demonstrated in which *M*. *schreibersii* bats from Hainan were actually *M*. *fuliginosus*^10,55^. This is due to the cryptic nature of several Miniopterus bat species. Distinctly, the identification based on their morphology is not sufficient, and typically requires the use of DNA barcoding or echolocation studies^56^.

Our results emphasized the evolution among PVs within bats horizontally, through host jumping mechanism, rather than co-speciation. Additionally, host specificity was not observed, suggesting it is not involved in the evolutionary history regarding BtPVs. Moreover, we found the occurrence of cross-species transmission events may likely increase with sympatry. Notwithstanding the exploitation of all available information in reference to BtPVs, admittedly, this study bears limitations, primarily related either to the quality of the available data, or the lack thereof. To cite an example, the precise classification of the Romanian putative mischiviruses was not possible due to the short length of the viral sequences. Additionally, we could not use several BtPVs sequences originating from America since the hosts’ species were unknown. Undeniably, the impact of the misclassification of several host species should not be entirely ignored.

Recombination occurrence was highlighted in this research and believed to play a part in both the diversity and the evolution regarding BtPVs, as aptly demonstrated in previous studies^57,58^. The phylogenetic resolution was affected by a lack of data reflected in polytomies observed in the virus phylogeny. Moreover, we noticed how the BtPVs data panel is small (n = 69) and unbalanced, since most studies were undertaken primarily throughout Europe and Asia, while other continents are characteristically, under-represented (America n = 1, Australia = 0). Furthermore, the sampled bats included in this study represent just 4.8% of the total currently recognized bat genera, and 2.2% of the total bat species known so far, leaving the greater majority yet unexplored.

Frankly speaking, our study is a starting point regarding further investigations in pursuit of the evolution of the PV family within these important flying mammals. Specifically, for this very purpose, we support additional sampling, detection and the acquisition of more BtPVs with longer sequences, including accurate host species identification.

## Supplementary information

Dataset 1
