# Supplementary material for: Genetic characterization of a novel picornavirus in Algerian bats: co-evolution analysis of bat-related picornaviruses
Source: Sci Rep. 2019 Oct 31;9:15706. doi: 10.1038/s41598-019-52209-2 (PMC6823487; doi:10.1038/s41598-019-52209-2)
Supplement: Supplementary file 1 — Dataset 1 [file 41598_2019_52209_MOESM1_ESM.pdf]

Genetic characterization of a novel picornavirus in Algerian bats: co-evolution analysis of bat-related picornaviruses

Safia Zeghbib, Robert Herczeg, Gábor Kemenesi, Brigitta Zana, Kornélia Kurucz, Peter Urbán, Mónika Madai, Fanni Földes, Henrietta Papp, Balázs Somogyi, Ferenc Jakab

PVs accession number	PVs genus	RdRp Length (bp)	Host Species	Final RdRp length (bp)	PVs sampling location
MG888045	Mischivirus	1403	*Miniopterus schreibersii*	432	Algeria
KJ641684	Unassigned (Bat picornavirus 2)	1317	*Rhinolophus ferrumequinum*	441	China
KT452742	Hepatovirus	1425	*Miniopterus manavi*	453	Madagascar
KT452730	Hepatovirus	1440	*Coleura afra*	456	Ghana
KT452729	Hepatovirus	1434	*Rhinolophus landeri*	456	Ghana
KT452714	Hepatovirus	1434	*Eidolon helvum*	456	Ghana
NC_033819	Crohivirus	1356	*Eidolon helvum*	411	Cameroon
KX644936	Kunsagivirus	1107	*Eidolon helvum*	414	Cameroon
641691	Unassigned (Kobu-like virus)	1350	*Miniopterus fuliginosus*	423	China
KJ641686	Kobuvirus	1341	*Myotis ricketti*	423	China
HM228882	(Kobu-like virus)	1198	*Eidolon helvum*	423	USA
KP100644	Mischivirus	1359	*Hipposideros gigas*	432	Congo
JQ814851	Mischivirus	1398	*Miniopterus schreibersii*	432	China
KP054278	Mischivirus	1401	*Miniopterus schreibersii*	432	Hungary
KP054273	Mischivirus	1401	*Miniopterus schreibersii*	432	Hungary
KP054276	Mischivirus	1401	*Miniopterus schreibersii*	432	Hungary
KP054275	Mischivirus	1401	*Miniopterus schreibersii*	432	Hungary
KP054274	Mischivirus	1401	*Miniopterus schreibersii*	432	Hungary
KP054277	Mischivirus	1401	*Miniopterus schreibersii*	432	Hungary
KJ641697	Unassigned (Sapelo-like)	1332	*Nyctalus plancyi*	420	China

KJ641685	Unassigned (Sapelo-like)	1344	*Rhinolophus ferrumequinum*	420	China
KJ641688	Unassigned (Sapelo-like)	1344	*Rhinolophus lepidus*	420	China
KJ641691	Kobuvirus	1350	*Miniopterus fuliginosus*	423	China
KJ641692	Unassigned (Sapelo-like)	1344	*Rhinolophus affinis*	420	China
KJ641698	Unassigned (Shanbavirus)	978	*Miniopterus fuliginosus*	414	China
KJ641689	Unassigned (Sapelo-like)	1335	*Myotis altarium*	414	China
KJ641696	Unassigned (Sapelo-like)	1335	*Vespertilio superans*	414	China
NC_033820	Unassigned (Sapelo-like)	1347	*Eidolon helvum*	420	Cameron
KJ641699	Unassigned (Sapelo-like)	1410	*Miniopterus fuliginosus*	420	China
KJ641687	Unassigned (Sapelo-like)	1410	*Miniopterus fuliginosus*	420	China
KJ641690	Unassigned (Sapelo-like)	1410	*Miniopterus fuliginosus*	420	China
HQ595343	Unassigned (bat picornavirus 2)	1383	*Miniopterus magnater*	456	Hong Kong
HQ595342	Unassigned (bat picornavirus 2)	1422	*Miniopterus magnater*	456	Hong Kong
HQ595341	Unassigned (bat picornavirus 1)	1416	*Miniopterus schreibersii*	450	Hong Kong
JQ814852	Unassigned (Ia io picornavirus 1)	1398	*Ia io*	420	China
KJ641694	Unassigned (Sapelo-like)	1344	*Rhinolophus sinicus*	420	China

KJ641695	Unassigned (Sapelo-like)	1344	*Rhinolophus sinicus*	420	China
KJ641693	Unassigned (bat picornavirus 3)	1344	*Rhinolophus hipposideros*	420	China
HQ595344	Unassigned (bat picornavirus 3)	1344	*Rhinolophus sinicus*	420	Hong Kong
HQ595345	Unassigned (bat picornavirus 3)	1383	*Hipposideros armiger*	420	Hong Kong
JQ916917	Unassigned (Sapelo-like)	744	*Rhinolophus blasii*	420	Bulgaria
JQ916918	Unassigned (Sapelo-like)	744	*Rhinolophus euryale*	420	Bulgaria
JQ916919	Unassigned (Enterovirus-G)	738	*Myotis bechsteini*	414	Germany
JQ916920	Unassigned (Sapelo-like)	744	*Rhinolophus euryale*	420	Bulgaria
JQ916921	Unassigned (Sapelo-like)	744	*Rhinolophus euryale*	420	Bulgaria
JQ916922	Unassigned (Ia io picornavirus)	744	*Myotis dasycneme*	420	germany
JQ916923	Unassigned (bat picornavirus 1-3)	744	*Miniopterus schreibersii*	450	Bulgaria
JQ916924	Unassigned (bat picornavirus 1-3)	744	*Miniopterus schreibersii*	450	Bulgaria
JQ916925	Unassigned (bat picornavirus 1-3)	744	*Miniopterus schreibersii*	450	Bulgaria
JQ916926	Unassigned (bat picornavirus 1-3)	744	*Miniopterus schreibersii*	450	Bulgaria

JQ916927	Unassigned (bat picornavirus 1-3)	744	*Miniopterus schreibersii*	450	Bulgaria
JQ916928	Unassigned (bat picornavirus 1-3)	744	*Miniopterus schreibersii*	450	Bulgaria
JQ916929	Unassigned Feline (picornavirus)	744	*Nyctalus noctula*	420	Romania
JQ916930	Unassigned Feline (picornavirus)	744	*Miniopterus schreibersii*	420	Romania
JQ916931	Unassigned (Pigeon picornavirus)	414	*Myotis myotis*	407	Germany
JQ916932	Unassigned (la io picornavirus)	420	*Myotis myotis*	420	Germany
JQ916933	Unassigned (la io picornavirus)	420	*Myotis myotis*	420	Germany
JQ916934	Unassigned (la io picornavirus)	420	*Myotis myotis*	420	Germany
JQ916935	Unassigned (Mischivirus C)	732	*Myotis myotis*	432	Romania
JQ916936	Unassigned (Mischivirus C)	732	Myotis myotis	432	Romania
JQ916937	Unassigned (Mischivirus C)	732	Myotis oxygnathus	432	Romania
JQ916938	Unassigned (Mischivirus B)	750	Miniopterus schreibersii	432	Bulgaria
JQ916939	Unassigned (Mischivirus B)	343	Miniopterus schreibersii	342	Bulgaria
JQ916940	Unassigned (Mischivirus B)	343	Miniopterus schreibersii	342	Bulgaria

JQ916941	Unassigned (Mischivirus B)	343	Miniopterus schreibersii	342	Bulgaria
JQ916942	Unassigned (Sapelo-like)	423	Rhinolophus ferrumequinum	411	Bulgaria
JQ916943	Unassigned (Sapelo-like)	420	Rhinolophus euryale	420	Bulgaria
JQ916944	Unassigned (Sapelo-like)	420	Rhinolophus euryale	420	Bulgaria
NC_015941	Unassigned (Bat picornavirus 2)	1422	Miniopterus magnater	456	Hong Kong
NC_015934	Unassigned (Bat picornavirus 3)	1383	Rhinolophus sinicus	420	Hong Kong
NC_027214	Ampivirus A	1446	Lissotriton vulgaris	506	Hungary

Supplementary Table S1. details of RdRp sequences used in this study. RdRp sequences used in this study, with their accession numbers, sampling locations, host species and initial and final length.

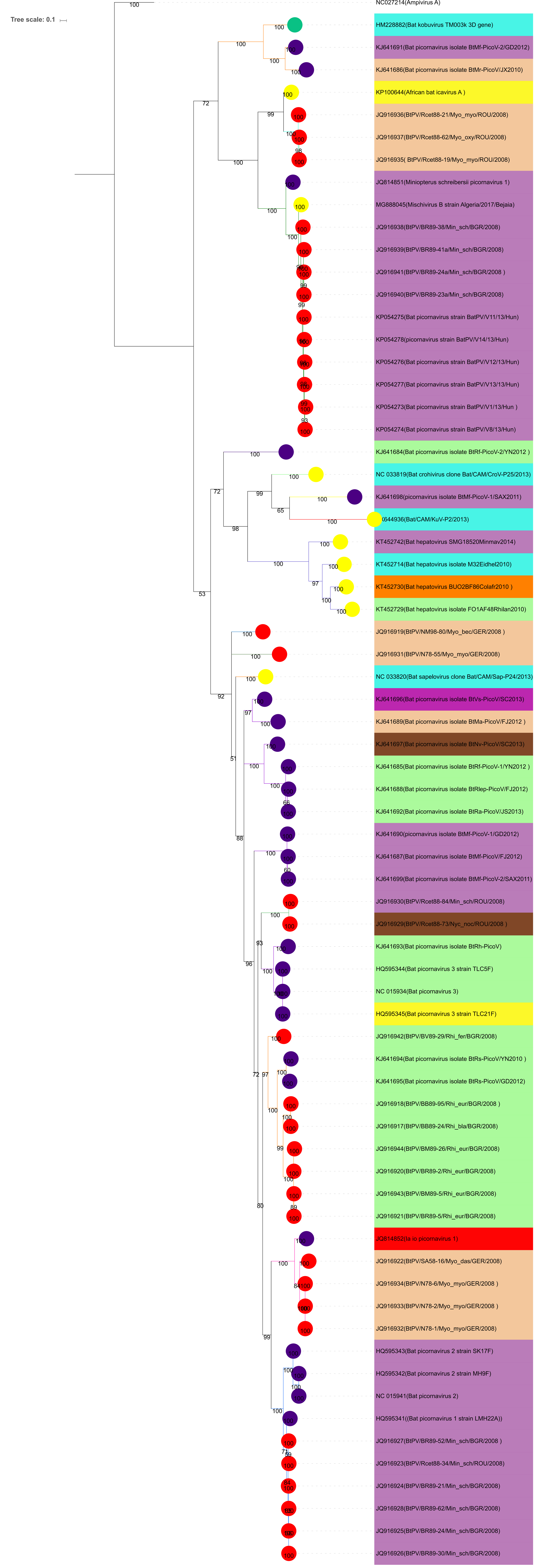

Supplementary Fig S1 A phylogenetic overview of PVs sequences analyzed. A Bayesian analysis of 70 RdRp sequences, rooted using Ampivirus A sequence (NC027214). Genus-specific clusters are colored based on bat genus. Filled circles indicate Large-scale sampling locations, red for Europe, purple for Asia yellow for Africa, light green for America. posterior probabilities values are indicated for each node.

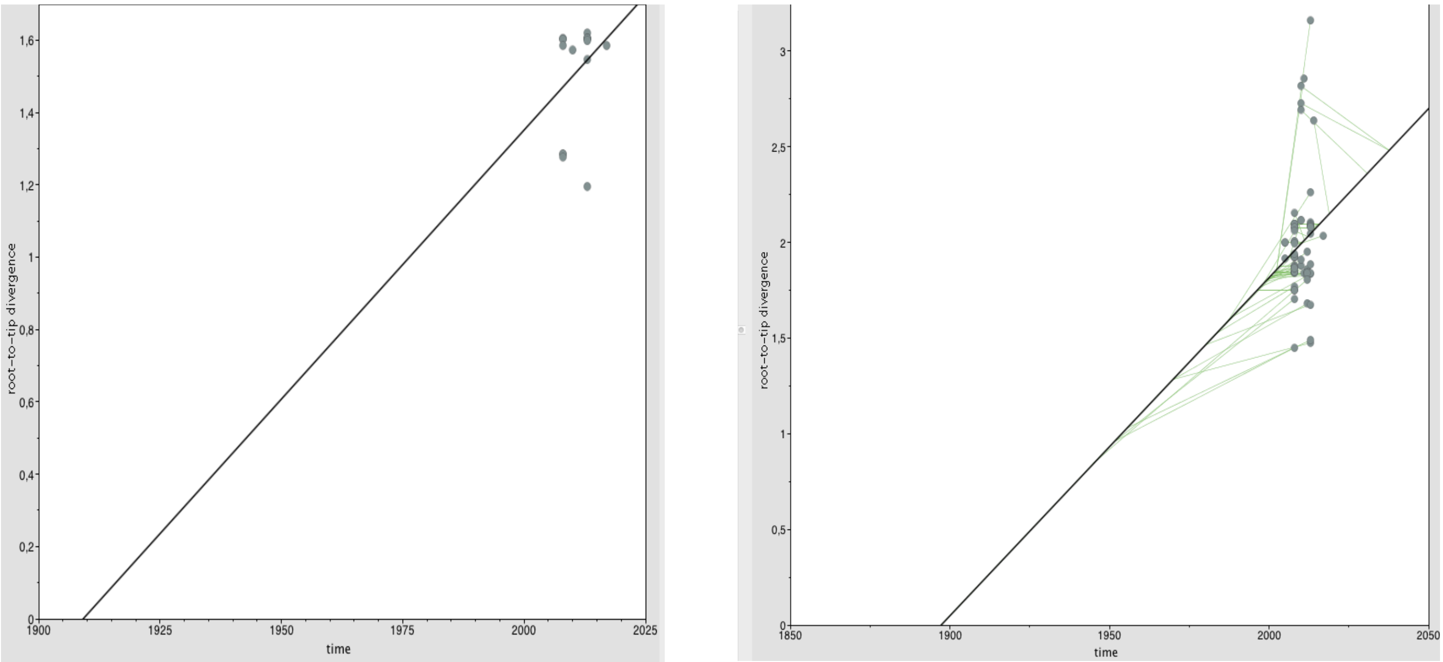

a Mischivirus

b All bat picornaviruses

Supplementary Fig S2. TempEst regression analysis between sampling times and root-to-tip genetic distances. The interpretation was based on R squared values. **a** bat mischiviruses R^2=0.0829. **b** all bat picornaviruses R^2=0.0218

Character trait	Statistics	Observed mean	Lower 95% CI	Upper 95% CU	Null mean	Lower 95% CI	Upper 95% CI	Significance
**Host genus**	AI	0.028	1.878E-5	0.031	0.746	0.344	1.122	0.0
	PS	2.00	2.0	2.0	4.562	3.995	5.0	0.0
	MC (Miniopterus)	11.999	12.0	12.0	3.300	1.966	5.836	9.999E-4
	MC (Hipposederos)	1.0	1.0	1.0	1.0	1.0	1.0	1.0
	MC (Myotis)	2.999	3.0	3.0	1.096	1.0	1.967	9.999E-4
**Host species**	AI	0.389	0.333	0.395	0.781	0.372	1.147	0.065
	PS	3.0	3.0	3.0	4.688	4.0	5.0	0.0
	MC (M. schreibersii)	11.999	12.0	12.0	3.211	1.964	5.836	9.999E-4
	MC (H. gigas)	1.0	1.0	1.0	1.0	1.0	1.0	1.0
	MC (M. oxygnathus)	1.0	1.0	1.0	1.0	1.0	1.0	1.0
	MC (M. myotis)	1.004	1.0	1.0	1.029	1.0	1.017	1.0
**Sampling location**	AI	0.029	$2.264E^{-4}$	0.031	0.622	0.211	1.022	0.003
	PS	3.0	3.0	3.0	3.735	3.0	4.0	0.235
	MC (Africa)	1.0	1.0	1.0	1.023	1.0	1.003	1.0
	MC (Europe)	9.996	10.0	10.0	3.822	2.036	5.875	0.009
	MC (Asia)	1.0	1.0	1.0	1.0	1.0	1.0	1.0

Supplementary Table S2. Phylogeny-trait association analysis of bat mischiviruses using BaTS.

Character trait	Statistics	Observed mean	Lower 95% CI	Upper 95% CU	Null mean	Lower 95% CI	Upper 95% CI	Significance
**Host genus**	AI	2.254	1.979	2.562	5.419	4.603	6.192	0.0
	PS	21.003	21.0	21.0	36.369	33.859	39.206	0.0
	MC (Miniopterus)	11.998	12.0	12.0	2.909	2.0	4.009	0.009
	MC (Myotis)	3.840	3.0	4.0	1.415	1.0	2.238	0.009
	MC (Rhinolophus)	8.966	9.0	9.0	1.822	1.0143	2.674	0.009
	MC (Nyctalus)	1.0	1.0	1.0	1.006	1.0	1.0	1.0
	MC (Hipposederos)	1.0	1.0	1.0	1.0	1.0	1.0	1.0
	MC (Eidolon)	1.0	1.0	1.0	1.0	1.0	1.0	1.0
	MC (Vespertilio)	1.0	1.0	1.0	1.0	1.0	1.0	1.0
	MC (Ia)	1.009	1.0	1.0	1.083	1.0	1.644	1.0
	MC (Coleura)	1.0	1.0	1.0	1.0	1.0	1.0	1.0
**Host species**	AI	3.479	3.163	3.856	6.750	6.152	7.214	0.0
	PS	32.988	33.0	33.0	47.595	45.728	49.726	0.0
	MC (Miniopterus schreibersii)	11.995	12.0	12.0	2.177	1.188	3.009	0.009
	MC (Myotis oxygnathus)	1.0	1.0	1.0	1.0	1.0	1.0	1.0
	MC (Myotis myotis)	2.998	3.0	3.0	1.124	1.0	1.894	0.009
	MC (Myotis bechsteinii)	1.0	1.0	1.0	1.0	1.0	1.0	1.0
	MC (Myotis dasycneme)	1.0	1.0	1.0	1.0	1.0	1.0	1.0
	MC (Rhinolophus euryale)	3.999	4.0	4.0	1.103	1.0	1.650	0.009
	MC (Rhinolophus blasii)	1.0	1.0	1.0	1.0	1.0	1.0	1.0
	MC (Rhinolophus ferrumequinum)	1.0	1.0	1.0	1.0	1.0	1.0	1.0
	MC (Nyctalus noctula)	1.0	1.0	1.0	1.0	1.0	1.0	1.0
	MC (Miniopterus magnater)	2.998	3.0	3.0	1.018	1.0	1.007	0.009
	MC (Ia io)	1.0	1.0	1.0	1.0	1.0	1.0	1.0
	MC (Rhinolophus sinicus)	1.999	2.0	2.0	1.052	1.0	1.254	0.009
	MC (Hipposederos armiger)	1.0	1.0	1.0	1.0	1.0	1.0	1.0
	MC (Rhinolophus hipposideros)	1.0	1.0	1.0	1.0	1.0	1.0	1.0
	MC (Miniopterus fuliginosus)	2.999	3.0	3.0	1.079	1.0	1.978	0.009
	MC (Rhinolophus lepidus)	1.0	1.0	1.0	1.0	1.0	1.0	1.0
	MC (Rhinolophus affinis)	1.0	1.0	1.0	1.012	1.0	1.0	1.0
	MC (Nyctalus plancyi)	1.0	1.0	1.0	1.0	1.0	1.0	1.0
	MC (Vespertilio superans)	1.0	1.0	1.0	1.0	1.0	1.0	1.0
	MC (Myotis altarium)	1.0	1.0	1.0	1.0	1.0	1.0	1.0
	MC (Eidolon helvum)	1.009	1.0	1.0	1.070	1.0	1.990	1.0
	MC (Myotis ricketti)	1.0	1.0	1.0	1.0	1.0	1.0	1.0
	MC (Hipposederos gigas)	1.0	1.0	1.0	1.0	1.0	1.0	1.0
	MC (Coleura afra)	1.0	1.0	1.0	1.0	1.0	1.0	1.0
	MC (Rhinolophus landeri)	1.0	1.0	1.0	1.0	1.0	1.0	1.0
	MC (Miniopterus manavi)	1.0	1.0	1.0	1.0	1.0	1.0	1.0
**Sampling location**	AI	0.603	0.476	0.944	4.551	3.711	5.361	0.0
	PS	15.027	14.0	16.0	29.587	26.641	32.001	0.0
	MC (Africa)	4.002	4.0	4.0	1.222	1.0	1.990	0.009
	MC (Europe)	9.926	10.0	10.0	3.416	2.000	4.999	0.009
	MC (Asia)	4.905	4.0	6.0	2.334	1.368	3.189	0.029
	MC (America)	1.0	1.0	1.0	1.0	1.0	1.0	1.0

Supplementary Table S5. Phylogeny-trait association analysis of all bat picornaviruses using BaTS**.**

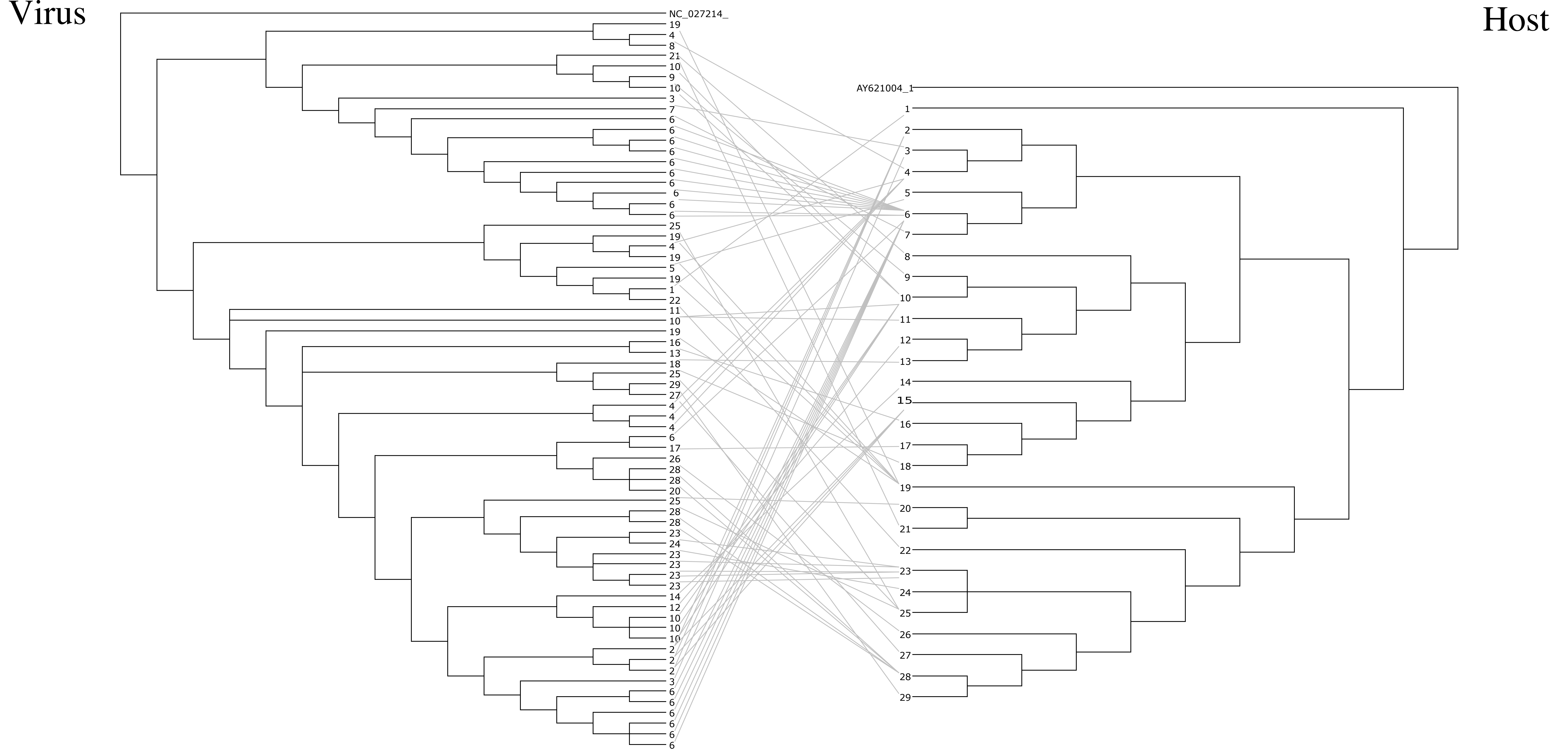

Supplementary Fig. S3. **Tanglegram for all bat picornaviruses and their hosts. NC_027214** *Ampivirus A* Virus phylogeny outgroup, **AY621004** *Furipterus horrens* host phylogeny outgroup, each number on the host tree indicates a bat species while, the same number on the virus tree indicates the bat species related viruses. *1: Coleura afra, 2: Miniopterus magnater, 3: Miniopterus schreibersii, 4: Miniopterus fuliginosus, 5: Miniopterus manavi, 6: M. schreibersii Hungary, 7: M.schreibersii Algeria, 8: Myotis ricketti, 9: Myotis oxygnathus, 10: Myotis myotis, 11: Myotis bechsteini, 12: Myottis dasycneme, 13: Myotis altarium, 14: Ia io, 15: Pipistrellus abramus, 16: Vespertilio superans, 17: Nyctalus noctule, 18: Nyctalus plancyi, 19: Eidolon helvum, 20: Hipposideros armiger, 21: Hipposideros gigas, 22: Rhinolophus landeri, 23: Rhinolophus euryale, 24: Rhinolophus blasii, 25: Rhinolophus ferrumequinum, 26: Rhinolophus hipposideros, 27: Rhinolophus affinis, 28: Rhinolophus sinicus, 29: Rhinolophus Lepidus*
